# Quantum Statistical Manifolds

**DOI:** 10.3390/e20060472

**Published:** 2018-06-17

**Authors:** Jan Naudts

**Affiliations:** Departement Fysica, Universiteit Antwerpen, Universiteitsplein 1, 2610 Wilrijk Antwerpen, Belgium; jan.naudts@uantwerpen.be

**Keywords:** quantum states, exponential connection, parameter-free information geometry, Banach manifold, GNS-representation

## Abstract

Quantum information geometry studies families of quantum states by means of differential geometry. A new approach is followed with the intention to facilitate the introduction of a more general theory in subsequent work. To this purpose, the emphasis is shifted from a manifold of strictly positive density matrices to a manifold of faithful quantum states on the C*-algebra of bounded linear operators. In addition, ideas from the parameter-free approach to information geometry are adopted. The underlying Hilbert space is assumed to be finite-dimensional. In this way, technicalities are avoided so that strong results are obtained, which one can hope to prove later on in a more general context. Two different atlases are introduced, one in which it is straightforward to show that the quantum states form a Banach manifold, the other which is compatible with the inner product of Bogoliubov and which yields affine coordinates for the exponential connection.

## 1. Introduction

The basic example of a quantum statistical system starts with a self-adjoint operator *H* on a finite-dimensional or separable Hilbert space H, with the property that the operator exp(−βH) is trace-class for all β in an open interval *D* of the real line R. Then, for β∈D, the quantum expectation value ${\langle A\rangle }_{\beta }$ of any bounded operator *A* in H is given by

(1) $$\begin{array}{ccc} {\langle A\rangle }_{\beta } & = & \frac{1}{Z(\beta )}\;\mathrm{Tr}\;e^{-\beta H}A,\;with\;Z\left(\beta \right)=\;\mathrm{Tr}\;e^{-\beta H}. \end{array}$$

Note that the quantum expectation is well-defined because the product of a trace-class operator with a bounded operator is again a trace-class operator. The operator *H* is the *Hamiltonian*. It defines a one parameter family of quantum states via Equation (1).

The quantum state (1) is a simple example of a model belonging to the *quantum exponential family*. In this case, the quantum states form a one-dimensional manifold. The goal of the present work is to search for a quantum exponential family formulated in a parameter-free way, similar to the formulation of Pistone and coworkers [1,2,3,4] in the non-quantum case, and to investigate a further generalization involving deformed exponential functions along the lines set out by the author in [5,6]. An alternative approach to parameter-free quantum information geometry is described in [7]. Which approach eventually will lead to a fully developed theory is hard to predict. Such a theory is expected to affect several domains of research, including Quantum Information Theory, Statistical Physics, in particular the study of phase transitions, and Complexity Theory. For a recent review of Information Geometry applied to complexity, see [8].

Early efforts to use geometric methods in the study of non-commutative information theory include the work by Ingarden and coworkers. See, for instance, [9,10]. The relation with Amari’s Information Geometry [11,12] was studied by Hasegawa [13,14,15]. He introduced an alpha-family of divergences $D_{\alpha }\left(\rho ,\sigma \right)$ where ρ and σ are any pair of density operators on a finite-dimensional Hilbert space H. The approach relies strongly on the properties of the trace.

The metric on the manifold of density matrices is the scalar product introduced by Bogoliubov and used by Kubo and Mori in the context of linear response theory. The generalization to an inner product for vector states on a von Neumann algebra is given in [16]. See also [17].

A more recent account on quantum information geometry is found in Chapter 7 of [12]. See also [18,19] and Example 3.8 of [20].

The parameter-free approach of Pistone and coworkers was generalized to the quantum context by Grasselli and Streater [21,22,23,24], See also [25]. Both the classical case and the quantum case need a regularizing condition on the allowed density functions, respectively density operators. Under this condition, they form a Banach manifold. Recently, Newton [26,27] proposed an alternative regularization based on a specific choice of a deformed logarithmic function. Part of the arguments in [27] can be transposed to the quantum setting [28].

The structure of the paper is as follows. In the next section, quantum states are labeled with operators belonging to the commutant of the GNS-representation rather than with density matrices. Section 3 describes the plane tangent at the reference state. Next, an atlas is introduced which contains a multitude of charts, one for each element of the manifold. Theorem 4 proves that the manifold is a Banach manifold and that the cross-over maps are linear operators. Section 5 introduces the inner product of Bogoliubov. The metric tensor is calculated. Next, alternative charts are introduced and their relation with the metric tensor is investigated. Section 8 and Section 9 discuss the mixture and the exponential connections. Proposition 4 proves that the alternative charts provide affine coordinates for the exponential connection. Section 10 contains a short presentation of the additional structure provided in quantum information geometry by the existence of modular automorphism groups. The final section discusses the results obtained so far. An Appendix about the GNS-representation and the modular operator is added for convenience of the reader.

## 2. Representation Theorems

In the present paper, the Hilbert space H is assumed to be finite dimensional. This solves the question of choosing an appropriate topology on the manifold of quantum states. In addition, all operators under consideration are bounded continuous. In fact, they are finite-dimensional matrices. In this way, the technical difficulties of working with unbounded operators are avoided.

A density matrix ρ is a self-adjoint operator with discrete spectrum consisting of non-negative eigenvalues which add up to one. This implies that the trace satisfies Trρ=1. The operator $e^{-\beta H}/Z\left(\beta \right)$, mentioned in the Introduction, is a density matrix of the kind we have in mind.

Introduce the notation A=B(H) for the $C^{*}$-algebra of bounded linear operators on the Hilbert space H. The notion of a quantum state coincides with the notion of a (mathematical) state ω on A. The latter is defined as a linear functional ω:A↦C, which satisfies the conditions of positivity and of normalization

(2) $$\begin{array}{c} \omega (A^{*}A)\geq 0\;\mathrm{for}\mathrm{all}\;A\in A,\\ \omega (I)=1, \end{array}$$

where I is the identity operator and $A^{*}$ is the adjoint of *A*. In particular, any state ω belongs to the dual space of A as a Banach space.

The state ω is said to be faithful if $\omega (A^{*}A)=0$ implies A=0.

The Gelfand–Naimark–Segal (GNS) construction shows that given a state ω on a $C^{*}$-algebra A there exists a *-representation π of A as bounded linear operators on a Hilbert space $H_{\omega }$, together with an element Ω of $H_{\omega }$ such that

(3) $$\begin{array}{ccc} \omega (A) & = & (\pi (A)\Omega ,\Omega )\;\mathrm{for}\mathrm{all}\;A\in A \end{array}$$

and π(A) is dense in $H_{\omega }$. This representation is unique up to unitary equivalence. This representation is used here to make the transition from a situation where quantum states are described by a density matrix to the more general context of an arbitrary von Neumann algebra A of bounded operators on a separable Hilbert space H, together with a cyclic and separating vector Ω∈H of norm one.

In the case of the algebra of all *N*-by-*N* matrices a simple and explicit realization of the GNS-representation is possible. See the Appendix.

The relation between a density matrix ρ and the corresponding quantum state $\omega_{\rho }$, defined by

(4) $$\begin{array}{ccc} \omega_{\rho }\left(A\right) & = & \;\mathrm{Tr}\;\rho A\;\mathrm{for}\mathrm{all}\;A\in A \end{array}$$

is a one-to-one relation. Indeed, if two density matrices $\rho_{1}$ and $\rho_{2}$ produce the same quantum expectations, then they coincide. Conversely, because the Hilbert space H is finite-dimensional, any quantum state ω determines a density matrix ρ such that $\omega =\omega_{\rho }$. The state $\omega_{\rho }$ is faithful if and only if the density matrix ρ is strictly positive.

For the sake of completeness, the proof of the following result is reproduced.

**Theorem** **1.** *Let ρ and σ be two strictly positive density matrices operating in a finite-dimensional Hilbert space H. Let A denote the von Neumann algebra of linear operators on H. Let $\pi_{\rho },H_{\rho },\Omega_{\rho }$ be the GNS-representation induced by ρ. Then, there exists a unique strictly positive operator X in the commutant $\pi {\left(A\right)}^{\prime }$ such that*

(5) $$\begin{array}{ccc} \;\mathrm{Tr}\;\sigma A & = & (\pi_{\rho }\left(A\right)X^{1/2}\Omega_{\rho },X^{1/2}\Omega_{\rho })\;for\;all\;A\in A. \end{array}$$

**Proof.**  Because $H_{\rho }$ is finite-dimensional and $\Omega_{\rho }$ is cyclic and separating one has $\pi_{\rho }\left(A\right)\Omega_{\rho }=\pi_{\rho }{\left(A\right)}^{\prime }\Omega_{\rho }=H_{\rho }$. Hence, there exists *X* in $\pi_{\rho }{\left(A\right)}^{\prime }$ such that

$$\begin{array}{ccc} X\Omega_{\rho } & = & \pi_{\rho }\left(\sigma \rho^{-1}\right)\Omega_{\rho }. \end{array}$$

Then, one has for all A∈A

(6) $$\begin{array}{ccc} \left(\pi_{\rho }\left(A\right)\Omega_{\rho },X\Omega_{\rho }\right) & = & \left(\pi_{\rho }\left(A\right)\Omega_{\rho },\pi_{\rho }\left(\sigma \rho^{-1}\right)\Omega_{\rho }\right)\\ & = & \left(\pi_{\rho }\left(\rho^{-1}\sigma A\right)\Omega_{\rho },\Omega_{\rho }\right)\\ & = & \;\mathrm{Tr}\;\rho \rho^{-1}\sigma A\\ & = & \;\mathrm{Tr}\;\sigma A. \end{array}$$

In particular, take $A=B^{*}B$ to obtain

(7) $$\begin{array}{ccc} \left(X\pi_{\rho }\left(B\right)\Omega_{\rho },\pi_{\rho }\left(B\right)\Omega_{\rho }\right) & = & \;\mathrm{Tr}\;\sigma B^{*}B\\ & \geq 0, \end{array}$$

with equality if and only if B=0. This implies that *X* is a strictly positive operator.

$X\Omega_{\rho }$ is the unique element of $H_{\rho }$ for which Equation (6) holds. Because $\Omega_{\rho }$ is cyclic for $\pi_{\rho }$, it is separating for the commutant. Hence, *X* is unique as well. ☐

Introduce the notation $B_{\rho }$ for the real Banach space formed by the self-adjoint elements *K* of $\pi_{\rho }{\left(A\right)}^{\prime }$ satisfying $(K\Omega_{\rho },\Omega_{\rho })=0$.

**Theorem** **2.** *Let H, A and ρ be as in the previous Theorem. Let $\pi_{\rho },H_{\rho },\Omega_{\rho }$ the GNS-representation induced by ρ. There is a one-to-one correspondence $\xi_{\rho }$ between faithful states ω on A and elements of $B_{\rho }$. It satisfies*

(8) $$\begin{array}{ccc} \omega (A) & = & e^{-\alpha_{\rho }\left(K\right)}\left(\pi_{\rho }\left(A\right)e^{\frac{1}{2}K}\Omega_{\rho },e^{\frac{1}{2}K}\Omega_{\rho }\right)\;for\;all\;A\in A, \end{array}$$

*with $K=\xi_{\rho }\left(\omega \right)$ and the function $\alpha_{\rho }$ given by*

$$\begin{array}{ccc} \alpha_{\rho }\left(K\right) & = & \log(e^{K}\Omega_{\rho },\Omega_{\rho }). \end{array}$$

**Proof.**  Let $\omega =\omega_{\sigma }$. The previous theorem guarantees the existence of a unique strictly positive operator *X* in the commutant $\pi_{\rho }{\left(A\right)}^{\prime }$. This operator *X* can be exponentiated. Let

$$\begin{array}{c} K=\log X-(\log X\Omega_{\rho },\Omega_{\rho }). \end{array}$$

Then, $(K\Omega_{\rho },\Omega_{\rho })=0$ holds by construction and Equation (8) is satisfied with $\alpha_{\rho }\left(K\right)=-\left(\log X\Omega_{\rho },\Omega_{\rho }\right)$ (remember that $(X\Omega_{\rho },\Omega_{\rho })=1$).

Conversely, given *K*, the r.h.s. of Equation (8) defines a faithful state ω of A. ☐

The map $\xi_{\rho }$ is a chart which makes the manifold M of all faithful quantum states into a Banach manifold. The chart $\xi_{\rho }$ is said to be *centered* at ρ. It satisfies $\xi_{\rho }\left(\rho \right)=0$.

All representations $\pi_{\rho }$, $H_{\rho }$, with ρ strictly positive, are unitary equivalent and can be identified. Therefore, in what follows, the index ρ of $\pi_{\rho }$ is dropped and the Hilbert space in which the representation π works is denoted $H_{\pi }$.

## 3. The Tangent Plane at the Center

Let $K=\xi_{\rho }\left(\sigma \right)\in B_{\rho }$. Introduce the notation

$$\begin{array}{ccc} \Psi_{K} & = & e^{\frac{1}{2}\left(K-\alpha \left(K\right)\right)}\Omega_{\rho }. \end{array}$$

One has

(9) $$\begin{array}{ccc} \frac{\;d\;}{\;dt}{|}_{t=0}\alpha_{\rho }\left(tK\right) & = & \frac{\left(Ke^{tK}\Omega_{\rho },\Omega_{\rho }\right)}{\left(e^{tK}\Omega_{\rho },\Omega_{\rho }\right)}{|}_{t=0}\\ & = & \left(K\Omega_{\rho },\Omega_{\rho }\right)\\ & = & 0 \end{array}$$

and

$$\begin{array}{ccc} \frac{\;d\;}{\;dt}{|}_{t=0}\Psi_{tK} & = & \frac{\;d\;}{\;dt}{|}_{t=0}e^{\frac{1}{2}\left(tK-\alpha \left(tK\right)\right)}\Omega_{\rho }\\ & = & \frac{1}{2}K\Omega_{\rho }. \end{array}$$

The density matrix $\sigma_{t}$, defined by

$$\begin{array}{ccc} \;\mathrm{Tr}\;\sigma_{t}A & = & (\pi \left(A\right)\Psi_{tK},\Psi_{tK}),\;A\in A \end{array}$$

satisfies

$$\begin{array}{ccc} \frac{\;d\;}{\;dt}{|}_{t=0}\;\mathrm{Tr}\;\sigma_{t}A & = & (\pi \left(A\right)K\Omega_{\rho },\Omega_{\rho }). \end{array}$$

Hence, the linear functional $f_{\rho ,K}$ defined by

(10) $$\begin{array}{c} A\in A\mapsto f_{\rho ,K}\left(A\right)=\left(\pi \left(A\right)\Omega_{\rho },K\Omega_{\rho }\right) \end{array}$$

is the derivative of the quantum state $\omega_{\rho }$ in the direction $\omega_{\sigma }$, where the density matrix $\sigma =\sigma_{1}$ has the property that $\xi_{\rho }\left(\sigma \right)=K$.

One concludes that the tangent plane $T_{\rho }M$ at the point $\omega_{\rho }\in M$ consists of all linear hermitian functionals $f_{\rho ,K}:\;A\mapsto C$ of the form (10), with $K\in B_{\rho }$. The functional $f_{\rho ,K}$ belongs to the dual of A. In addition,

$$\begin{array}{ccc} \left|\right|f_{\rho ,K}\left|\right| & = & \underset{A\in A}{\sup}\left\{f_{\rho ,K}\left(A\right):\;||A||\leq 1\right\}\\ & = & \underset{A\in A}{\sup}\left\{(\pi \left(A\right)K\Omega_{\rho },\Omega_{\rho }):\;||A||\leq 1\right\}\\ & = & \left|\right|{\left|K\right|}^{1/2}\Omega_{\rho }{\left|\right|}^{2}\\ & \leq & \left|\right|{\left|K\right|}^{1/2}{\left|\right|}^{2}=||K||. \end{array}$$

Hence, $K\mapsto f_{\rho ,K}$ is a bounded linear operator. This is a prerequisite for proving in the next Theorem that this map is the Fréchet derivative of the inverse of the chart $\xi_{\rho }$. This bounded operator is denoted $F_{\rho }$ in what follows. One has $F_{\rho }K=f_{\rho ,K}$. The inverse operator $F_{\rho }^{-1}$ satisfies $F_{\rho }^{-1}f_{\rho ,K}=K$. It is well-defined. Indeed, $f_{\rho ,K}=f_{\rho ,L}$ implies for all B∈A

$$\begin{array}{ccc} 0 & = & \left(\pi \left(B^{*}B\right)\left(K-L\right)\Omega_{\rho },\Omega_{\rho }\right)\\ & = & (\left(K-L\right)\pi \left(B\right)\Omega_{\rho },\pi \left(B\right)\Omega_{\rho }). \end{array}$$

Because $\Omega_{\rho }$ is a cyclic vector it follows that K=L.

**Theorem** **3.** The inverse of the map $\xi_{\rho }:\;M\mapsto B_{\rho }$, defined in Theorem 2, is Fréchet-differentiable at $\omega =\omega_{\rho }$. The Fréchet derivative is denoted $F_{\rho }$. It maps K to $f_{\rho ,K}$, where the latter is defined by Expression (10).

**Proof.**  Let $K=\xi_{\rho }\left(\omega_{\sigma }\right)$. One calculates

(11) $$\begin{array}{ccc} \left|\right|\omega_{\sigma }-\omega_{\rho }-F_{\rho }K\left|\right| & = & \underset{A\in A}{\sup}\left\{|\omega_{\sigma }\left(A\right)-\omega_{\rho }\left(A\right)-F_{\rho }K\left(A\right)|:\;||A||\leq 1\right\}\\ & = & \underset{A\in A}{\sup}\left\{|(\pi \left(A\right)\Omega_{\rho },\left[e^{K-\alpha (K)}-I-K\right]\Omega_{\rho })|:\;||A||\leq 1\right\}\\ & \leq & \left|\right|e^{K-\alpha (K)}-I-K\left|\right|\\ & \leq & |\alpha (K)|+o(||K-\alpha (K)||). \end{array}$$

Note that

$$\begin{array}{c} \left|\alpha \left(K\right)\right|\leq \log||e^{K}\left|\right|\leq \left||K|\right| \end{array}$$

and

(12) $$\begin{array}{c} ||K-\alpha (K)||\leq 2||K||. \end{array}$$

In addition, if ||K|| < 1 then one has

$$\alpha_{\rho }(K)\leq \text{log}(1+||K\Omega_{\rho }|{|}^{2})\leq |K\Omega_{\rho }|{|}^{2}.$$

This holds because λ ≤ 1 implies exp(λ) ≤ 1 + λ + λ^2^. One concludes that (11) converges to 0 faster than linearly as ||K|| tends to 0. This proves that $F_{\rho }K$ is the Fréchet derivative of $\xi_{\rho }\left(\omega_{\sigma }\right)\mapsto \omega_{\sigma }$ at σ=ρ. ☐

## 4. The Atlas

Following the approach of Pistone and collaborators [1,3,4,27], we build an atlas of charts $\xi_{\rho }$, one for each strictly positive density matrix ρ. The compatibility of the different charts requires the study of the cross-over map $\xi_{\rho_{1}}\left(\sigma \right)\mapsto \xi_{\rho_{2}}\left(\sigma \right)$, where $\rho_{1},\rho_{2},\sigma$ are arbitrary strictly positive density matrices.

Simplify notations by writing $\xi_{1}$ and $\xi_{2}$ instead of $\xi_{\rho_{1}}$, respectively $\xi_{\rho_{2}}$. Similarly, write $\Omega_{1}$ and $\Omega_{2}$ instead of $\Omega_{\rho_{1}}$, respectively $\Omega_{\rho_{2}}$, and $F_{1},F_{2}$ instead of $F_{\rho_{1}}$, respectively $F_{\rho_{2}}$.

Continuity of the cross-over map follows from the continuity of the exponential and logarithmic functions and from the following result.

**Proposition** **1.** Fix strictly positive density matrices $\rho_{1}$ and $\rho_{2}$. There exists a linear operator Y such that for any strictly positive density matrix σ and corresponding positive operators $X_{1}$, $X_{2}$ in the commutant $A^{\prime }$ one has $X_{2}=YX_{1}Y^{*}$.

**Proof.**  Using the notations of the Appendix one has

$$\begin{array}{ccc} X_{i} & = & J_{i}\left(\rho_{i}^{-1/2}\sigma \rho_{i}^{-1/2}⊗I\right)J_{i}^{*},\;i=1,2. \end{array}$$

Note that the isometry *J* depends on the reference state with density matrix ρ. Therefore it carries an index *i*. The above expression for $X_{i}$ implies that

$$\begin{array}{c} X_{2}=YX_{1}Y^{*}\;with\;Y=J_{2}\left(\rho_{2}^{-1/2}\rho_{1}^{1/2}⊗I\right)J_{1}^{*}. \end{array}$$

☐

**Theorem** **4.** *The set M of faithful states on the algebra A of square matrices, together with the atlas of charts $\xi_{\rho }$, where $\xi_{\rho }$ is defined by Theorem 1, is a Banach manifold. For any pair of strictly positive density matrices $\rho_{1}$ and $\rho_{2}$, the cross-over map $\xi_{2}\circ \xi_{1}^{-1}$ is continuous.*

**Proof.**  The continuity of the map $X_{1}\mapsto X_{2}$ follows from the previous Proposition. The continuity of the maps $K_{1}\mapsto X_{1}$ and $X_{2}\mapsto K_{2}$ follows from the continuity of the exponential and logarithmic functions and the continuity of the function α. ☐

## 5. The Bogoliubov Inner Product

Umegaki’s divergence/relative entropy D(σ,τ) of a pair of strictly positive density matrices σ and τ is defined by [29,30,31]

$$\begin{array}{ccc} D(\sigma ||\tau ) & = & \;\mathrm{Tr}\;\sigma (\log\sigma -\log\tau ). \end{array}$$

It can be used to define a metric tensor $g_{\sigma ,\tau }\left(\rho \right)$, as explained below.

In the commutative context, Chentsov proved the uniqueness of the Fisher information matrix as a metric which is invariant under Markov morphisms. See, for instance, Theorem 2.1 of [32]. In the quantum case, the additional requirement of the existence of a dually-flat geometry is needed [21]. The notion of quantum relative entropy comes from Quantum Statistical Physics. In Quantum Information Theory, other quantities are being used as well. Alternatives include the trace distance, the Bures distance and the related fidelity function. See, for instance, Chapter 6 of [20].

Introduce $\sigma_{s}$ and $\tau_{t}$ given by

(13) $$\begin{array}{ccc} \sigma_{s} & = & \frac{1}{Z(s)}\exp\left(\log\rho +s(\log\sigma -\log\rho )\right),\\ \tau_{t} & = & \frac{1}{W(t)}\exp\left(\log\rho +t(\log\tau -\log\rho )\right), \end{array}$$

with

(14) $$\begin{array}{ccc} Z(s) & = & \;\mathrm{Tr}\;\exp\left(\log\rho +s(\log\sigma -\log\rho )\right),\\ W(t) & = & \;\mathrm{Tr}\;\exp\left(\log\rho +t(\log\tau -\log\rho )\right). \end{array}$$

Both $\sigma_{s}$ and $\tau_{t}$ are well-defined density matrices. The maps $s\mapsto \omega_{\sigma_{s}}$ and $t\mapsto \omega_{\tau_{t}}$ describe two orbits in M, intersecting at $\omega_{\rho }$: $\sigma_{0}=\tau_{0}=\rho$. For further use, note that Z(0)=W(0)=Z(1)=W(1)=1.

The metric tensor $g_{\sigma ,\tau }\left(\rho \right)$ is defined by

(15) $$\begin{array}{ccc} g_{\sigma ,\tau }\left(\rho \right) & = & -\frac{\partial \;}{\partial s}\frac{\partial \;}{\partial t}{|}_{s=t=0}D(\sigma_{s}\left|\right|\tau_{t}). \end{array}$$

With the help of the identity

$$\begin{array}{ccc} \frac{\;d\;}{\;dt}{|}_{t=0}e^{H+tA} & = & \int_{0}^{1}\;du\;e^{uH}Ae^{(1-u)H}, \end{array}$$

one obtains

$$\begin{array}{ccc} \frac{\;d\;}{\;dt}{|}_{t=0}\log\tau_{t} & = & \log\tau -\log\rho -\frac{\;d\;}{\;dt}{|}_{t=0}\log W\left(t\right)\\ & = & \log\tau -\log\rho -\int_{0}^{1}\;du\;\;\mathrm{Tr}\;\rho^{u}\left(\log\tau -\log\rho \right)\rho^{1-u}\\ & = & \log\tau -\log\rho +D(\rho ||\tau ), \end{array}$$

so that

$$\begin{array}{ccc} g_{\sigma ,\tau }\left(\rho \right) & = & \frac{\partial \;}{\partial s}\frac{\partial \;}{\partial t}{|}_{s=t=0}\;\mathrm{Tr}\;\sigma_{s}\log\tau_{t}\\ & = & \frac{\;d\;}{\;ds}{|}_{s=0}\;\mathrm{Tr}\;\sigma_{s}\left[\log\tau -\log\rho +D(\rho ||\tau )\right]\\ & = & \frac{\;d\;}{\;ds}{|}_{s=0}\;\mathrm{Tr}\;\sigma_{s}\left[\log\tau -\log\rho \right]\\ & = & \int_{0}^{1}\;du\;\;\mathrm{Tr}\;\rho^{u}\left(\log\sigma -\log\rho \right)\rho^{1-u}\left[\log\tau -\log\rho )\right]\\ & & -\left(\frac{\;d\;}{\;ds}{|}_{s=0}Z\left(s\right)\right)\;\mathrm{Tr}\;\rho \left[\log\tau -\log\rho \right]\\ & = & \int_{0}^{1}\;du\;\;\mathrm{Tr}\;\rho^{u}\left(\log\sigma -\log\rho \right)\rho^{1-u}\left(\log\tau -\log\rho \right)\\ & & -D(\rho ||\sigma )D(\rho ||\tau ). \end{array}$$

This is the inner product of Bogoliubov. Its positivity is shown in the next section. It is straightforward to check that $g_{\sigma ,\tau }=g_{\tau ,\sigma }$.

## 6. Alternative Charts

The inner product (16) is expressed in terms of density matrices rather than tangent vectors. Let us therefore calculate the tangent vector of the orbit $\omega_{\sigma_{s}}$ defined by Definition (13).

**Lemma** **1.** *For each A, self-adjoint element of A such that $\omega_{\rho }\left(A\right)=0$, there exists a unique element K of $B_{\rho }$ such that*

(17) $$\begin{array}{ccc} \int_{0}^{1}\;du\;\pi \left(\rho^{u}A\rho^{-u}\right)\Omega_{\rho } & = & K\Omega_{\rho }. \end{array}$$

**Proof.**  An operator *K* in the commutant $\pi {\left(A\right)}^{\prime }$ satisfying Equation (17) exists because $\pi {\left(A\right)}^{\prime }\Omega_{\rho }=H_{\pi }$. It is unique because $\Omega_{\rho }$ is separating for $\pi {\left(A\right)}^{\prime }$. It satisfies

$$\begin{array}{ccc} \left(K\Omega_{\rho },\Omega_{\rho }\right) & = & \int_{0}^{1}\;du\;\left(\pi \left(\rho^{u}A\rho^{-u}\right)\Omega_{\rho },\Omega_{\rho }\right)\\ & = & \int_{0}^{1}\;du\;\;\mathrm{Tr}\;\rho^{1+u}A\rho^{-u}\\ & = & \omega_{\rho }\left(A\right)\\ & = & 0. \end{array}$$

Finally, for any *B* in A, one has

$$\begin{array}{ccc} \left(K^{*}\Omega_{\rho },\pi \left(B\right)\Omega_{\rho }\right) & = & \left(\pi \left(B^{*}\right)\Omega_{\rho },K\Omega_{\rho }\right)\\ & = & \left(\pi \left(B^{*}\right)\Omega_{\rho },\int_{0}^{1}\;du\;\pi \left(\rho^{u}A\rho^{-u}\right)\Omega_{\rho }\right)\\ & = & \int_{0}^{1}\;du\;\left(\pi \left(\rho^{-u}A\rho^{u}B^{*}\right)\Omega_{\rho },\Omega_{\rho }\right)\\ & = & \int_{0}^{1}\;du\;\;\mathrm{Tr}\;\rho^{1-u}A\rho^{u}B^{*}\\ & = & \int_{0}^{1}\;du\;\;\mathrm{Tr}\;\rho^{u}A\rho^{1-u}B^{*}\\ & = & \int_{0}^{1}\;du\;\left(\pi \left(B^{*}\rho^{u}A\rho^{-u}\right)\Omega_{\rho },\Omega_{\rho }\right)\\ & = & (K\Omega_{\rho },\pi \left(B\right)\Omega_{\rho }). \end{array}$$

This shows that $K=K^{*}$. One concludes that *K* belongs to $B_{\rho }$. ☐

**Lemma** **2.** *There exists a strictly positive operator $G_{\rho }$ on $H_{\pi },$ which satisfies*

$$\begin{array}{ccc} G_{\rho }\int_{0}^{1}\;du\;\pi \left(\rho^{u}A\rho^{-u}\right)\Omega_{\rho } & = & \pi \left(A\right)\Omega_{\rho }\;for\;all\;A\in A. \end{array}$$

**Proof.**  First, consider the operator *X* defined by

$$\begin{array}{ccc} X\pi \left(A\right)\Omega_{\rho } & = & \int_{0}^{1}\;du\;\pi \left(\rho^{u}A\rho^{-u}\right)\Omega_{\rho }. \end{array}$$

It is well-defined because $\pi \left(A\right)\Omega_{\rho }=0$ implies A=0. It is a positive operator. This follows from

$$\begin{array}{ccc} \left(X\pi \left(A\right)\Omega_{\rho },\pi \left(A\right)\Omega_{\rho }\right) & = & \int_{0}^{1}\;du\;\left(\pi \left(\rho^{u}A\rho^{-u}\right)\Omega_{\rho },\pi \left(A\right)\Omega_{\rho }\right)\\ & = & \int_{0}^{1}\;du\;\;\mathrm{Tr}\;\rho A^{*}\rho^{u}A\rho^{-u}\\ & = & \int_{0}^{1}\;du\;\;\mathrm{Tr}\;\rho^{(1-u)/2}A^{*}\rho^{u}A\rho^{(1-u)/2}\\ & \geq & 0. \end{array}$$

The latter expression vanishes if and only if $\rho^{(1-u)/2}A^{*}\rho^{u}A\rho^{(1-u)/2}=0$ for almost all *u* in [0,1]. Because ρ is strictly positive, this can happen only if A=0. This shows that the operator *X* is invertible. Take $G_{\rho }$ equal to the inverse of *X* to obtain the desired result. ☐

**Theorem** **5.** *Let H, A and ρ be as in the previous theorems. Let $\pi ,H_{\pi },\Omega_{\rho }$ be the GNS-representation of A induced by ρ. Let $G_{\rho }$ be the positive operator defined by the previous lemma.*

*i)*  *There exists a map $\chi_{\rho }$ from the faithful states ω on A into the real Banach space $B_{\rho }$, formed by the self-adjoint elements K of $\pi_{\rho }{\left(A\right)}^{\prime }$ satisfying $(K\Omega_{\rho },\Omega_{\rho })=0$, such that for any strictly positive density matrix σ one has*
  $$\begin{array}{ccc} G_{\rho }\chi_{\rho }\left(\omega_{\sigma }\right)\Omega_{\rho } & = & \pi \left(A_{\rho ,\sigma }\right)\Omega_{\rho }, \end{array}$$
  *with $A_{\rho ,\sigma }$ in A given by*
  $$\begin{array}{ccc} A_{\rho ,\sigma } & = & \log\sigma -\log\rho +D(\rho ||\sigma ). \end{array}$$
*ii)*  The map $\chi_{\rho }$ is injective.
*iii)*  *For each strictly positive density matrix σ is*
  $$\begin{array}{ccc} \frac{\;d\;}{\;ds}{|}_{s=0}\omega_{\sigma_{s}} & = & f_{\rho ,K}, \end{array}$$
  *with $K=\chi_{\rho }\left(\omega_{\sigma }\right)$, where $\sigma_{s}$ is defined by Definition (13) and $f_{\rho ,K}$ is defined by (10).*

**Proof.** 

i) By the previous lemma, one has $\pi \left(A_{\rho ,\sigma }\right)\Omega_{\rho }=G_{\rho }X\Omega_{\rho }$ with *X* defined by
  $$\begin{array}{ccc} X & = & \int_{0}^{1}\;du\;\pi \left(\rho^{u}A_{\rho ,\sigma }\rho^{-u}\right). \end{array}$$
  Note that $A_{\rho ,\sigma }$ is self-adjoint and satisfies $\omega_{\rho }\left(A_{\rho ,\sigma }\right)=0$. Hence, by Lemma 1, there exists a unique *K* in $B_{\rho }$ such that $X\Omega_{\rho }=K\Omega_{\rho }$. This shows that the map $\chi_{\rho }$ which maps $\omega_{\sigma }$ onto this element *K* of $B_{\rho }$ is well-defined.
ii) Assume that $\chi_{\rho }\left(\omega_{\sigma }\right)=\chi_{\rho }\left(\omega_{\tau }\right)$. This implies $A_{\rho ,\sigma }=A_{\rho ,\tau }$ and hence
  $$\begin{array}{ccc} \log\sigma +D(\rho ||\sigma ) & = & \log\tau +D(\rho ||\tau ). \end{array}$$
  The latter implies
  $$\begin{array}{ccc} \sigma & = & \tau e^{D(\rho ||\tau )-D(\rho ||\sigma )}. \end{array}$$
  Because Trσ=Trτ, it follows that D(ρ||τ)=D(ρ||σ) and hence σ=τ. This shows that the map $\chi_{\rho }$ is injective.
iii) One has for all B∈A
  $$\begin{array}{ccc} \frac{\;d\;}{\;ds}{|}_{s=0}\;\mathrm{Tr}\;\sigma_{s}B & = & D\left(\rho ||\sigma \right)\omega_{\rho }\left(B\right)+\int_{0}^{1}\;du\;\;\mathrm{Tr}\;\rho^{1-u}\left(\log\sigma -\log\rho \right)\rho^{u}B\\ & = & \int_{0}^{1}\;du\;\;\mathrm{Tr}\;\rho^{1-u}A_{\rho ,\sigma }\rho^{u}B\\ & = & \int_{0}^{1}\;du\;\;\mathrm{Tr}\;\rho^{1-u}B\rho^{u}A_{\rho ,\sigma }\\ & = & \int_{0}^{1}\;du\;\left(\pi \left(B\rho^{u}A_{\rho ,\sigma }\rho^{u}\right)\Omega_{\rho },\Omega_{\rho }\right)\\ & = & \left(\pi \left(B\right)G_{\rho }^{-1}\pi \left(A_{\rho ,\sigma }\right)\Omega_{\rho },\Omega_{\rho }\right)\\ & = & \left(\pi \left(B\right)\chi_{\rho }\left(\omega_{\sigma }\right)\Omega_{\rho },\Omega_{\rho }\right)\\ & = & f_{\rho ,K}\left(B\right), \end{array}$$
  with $K=\chi_{\rho }\left(\omega_{\sigma }\right)$.

☐

## 7. The Riemannian Metric

Introduce an inner product ${\langle \cdot ,\cdot \rangle }_{\rho }$ on $T_{\rho }M$ defined by

(18) $$\begin{array}{ccc} {\langle f_{\rho ,P},f_{\rho ,Q}\rangle }_{\rho } & = & \langle G_{\rho }P\Omega_{\rho },Q\Omega_{\rho }), \end{array}$$

for any P,Q in $B_{\rho }$. The matrix $G_{\rho }$, which is introduced in Lemma 2, is strictly positive. Hence, the inner product is positive and non-degenerate. It defines a Riemannian geometry on the manifold M.

**Theorem** **6.** *Let ρ,σ,τ be strictly positive density matrices. Let $\chi_{\rho }$ be the chart defined in Theorem 5. Let ${\langle \cdot ,\cdot \rangle }_{\rho }$ be the inner product defined on the tangent plane $T_{\rho }M$ by (18). The inner product of Bogoliubov, defined by (15), satisfies*

$$\begin{array}{ccc} g_{\sigma ,\tau }\left(\rho \right) & = & {\langle f_{\rho ,P},f_{\rho ,Q}\rangle }_{\rho }, \end{array}$$

*with $P=\chi_{\rho }\left(\sigma \right)$ and $Q=\chi_{\rho }\left(\tau \right)$.*

**Proof.**  From Expression (16) follows

$$\begin{array}{ccc} g_{\sigma ,\tau }\left(\rho \right) & = & \int_{0}^{1}\;du\;\left(\pi \left(\rho^{-u}\left(\log\tau -\log\rho \right)\rho^{u}\left(\log\sigma -\log\rho \right)\right)\Omega_{\rho },\Omega_{\rho }\right)\\ & & -D(\rho ||\sigma )D(\rho ||\tau )\\ & = & \int_{0}^{1}\;du\;\left(\pi \left(\rho^{-u}(A_{\rho ,\tau }-D(\rho ||\tau ))\rho^{u}(A_{\rho ,\sigma }-D(\rho ||\sigma ))\right)\Omega_{\rho },\Omega_{\rho }\right)\\ & & -D(\rho ||\sigma )D(\rho ||\tau ). \end{array}$$

Use now that

$$\begin{array}{c} \left(\pi \left(A_{\rho ,\sigma }\right)\Omega_{\rho },\Omega_{\rho }\right)=\int_{0}^{1}\;du\;\left(\pi \left(\rho^{-u}A_{\rho ,\tau }\rho^{u}\right)\Omega_{\rho },\Omega_{\rho }\right)=0 \end{array}$$

to obtain

$$\begin{array}{ccc} g_{\sigma ,\tau }\left(\rho \right) & = & \int_{0}^{1}\;du\;\left(\pi \left(\rho^{-u}\left(A_{\rho ,\tau }\right)\rho^{u}\left(A_{\rho ,\sigma }\right)\right)\Omega_{\rho },\Omega_{\rho }\right). \end{array}$$

This can be written as

$$\begin{array}{ccc} g_{\sigma ,\tau }\left(\rho \right) & = & \left(\pi \left(A_{\rho ,\sigma }\right)\Omega_{\rho },G_{\rho }^{-1}\pi \left(A_{\rho ,\tau }\right)\Omega_{\rho }\right)\\ & = & \left(G_{\rho }\chi_{\rho }\left(\sigma \right)\Omega_{\rho },\chi_{\rho }\left(\tau \right)\Omega_{\rho }\right)\\ & = & {\langle f_{\rho ,P},f_{\rho ,Q}\rangle }_{\rho }. \end{array}$$

☐

## 8. The Mixture Connection

Consider the situation in which the affine combinations of the form

$$\begin{array}{c} t\mapsto \rho_{t}=\left(1-t\right)\rho_{0}+t\rho_{1} \end{array}$$

are the geodesics of the geometry. Introduce the abbreviation $\omega_{t}=\omega_{\rho_{t}}$, with the latter defined by (4). The derivative

$$\begin{array}{ccc} \frac{\;d\;}{\;dt}\omega_{t} & = & \omega_{1}-\omega_{0} \end{array}$$

is a tangent vector, which is constant. This implies a vanishing connection.

## 9. The Exponential Connection

On the other hand, in the case of the exponential connection, the geodesics $t\mapsto \rho_{t}$ are such that

(19) $$\begin{array}{ccc} \log\rho_{t} & = & \left(1-t\right)\log\rho_{0}+t\log\rho_{1}-\zeta \left(t\right)\\ & = & \log\rho_{0}+tH-\zeta \left(t\right), \end{array}$$

where ζ(t) is a normalizing function and *H* is defined by $H=\log\rho_{1}-\log\rho_{0}$. Note that

(20) $$\begin{array}{ccc} \omega_{t}\left(H\right) & = & \;\mathrm{Tr}\;\rho_{t}H\\ & = & \;\mathrm{Tr}\;\rho_{t}\left[\log\rho_{1}-\log\rho_{0}\right]\\ & = & D(\rho_{t}\left|\right|\rho_{0})-D(\rho_{t}\left|\right|\rho_{1}). \end{array}$$

One has

$$\begin{array}{ccc} \frac{\;d\;}{\;dt}\rho_{t} & = & \frac{\;d\;}{\;d\epsilon }{|}_{\epsilon =0}\exp\left(\log\rho_{t}+\epsilon H\right)-\frac{\;d\zeta }{\;dt}\rho_{t}\\ & = & \int_{0}^{1}\;du\;{\left(\rho_{t}\right)}^{u}H{\left(\rho_{t}\right)}^{1-u}-\frac{\;d\zeta }{\;dt}\rho_{t}. \end{array}$$

Therefore, the derivative of the quantum state becomes

(21) $$\begin{array}{ccc} \frac{\;d\;}{\;dt}\omega_{t}\left(A\right) & = & \int_{0}^{1}\;du\;\;\mathrm{Tr}\;{\left(\rho_{t}\right)}^{u}H{\left(\rho_{t}\right)}^{1-u}A\\ & & -\frac{\;d\zeta }{\;dt}\omega_{t}\left(A\right). \end{array}$$

Take A=I to find that

(22) $$\begin{array}{c} \frac{\;d\zeta }{\;dt}=\omega_{t}\left(H\right). \end{array}$$

**Proposition** **2.** The function ζ(t) is convex.

**Proof.**  Let $A=\rho_{t}^{u/2}H\rho_{t}^{-u/2}$. Then, one has

$$\begin{array}{ccc} \;\mathrm{Tr}\;\rho_{t}^{1-u}H\rho_{t}^{u}H & = & \;\mathrm{Tr}\;\rho_{t}A^{*}A\\ & \geq & |\;\mathrm{Tr}\;\rho_{t}{A|}^{2}\\ & = & |\omega_{t}{\left(H\right)|}^{2}. \end{array}$$

Use this in

(23) $$\begin{array}{ccc} \frac{\;d^{2}\;}{\;dt^{2}}\zeta \left(t\right) & = & \frac{\;d\;}{\;dt}\;\mathrm{Tr}\;\rho_{t}H\\ & = & \int_{0}^{1}\;du\;\left[\;\mathrm{Tr}\;\rho_{t}^{1-u}H\rho_{t}^{u}H-{\left|\omega_{t}\left(H\right)\right|}^{2}\right]\\ & \geq & 0. \end{array}$$

☐

Because ζ(0)=ζ(1)=0, it follows that ζ(t)≤0 on 0<t<1. From Expression (20) and

$$\begin{array}{c} \frac{\;d\;}{\;dt}\omega_{t}\left(H\right)=\frac{\;d^{2}\;}{\;dt^{2}}\zeta \left(t\right)\geq 0, \end{array}$$

it follows that the expectation $\omega_{t}\left(H\right)$ increases from $\omega_{0}\left(H\right)=-D(\rho_{0}\left|\right|\rho_{1})\leq 0$ to $\omega_{1}\left(H\right)\;=\;D(\rho_{1}\left|\right|\rho_{0})\geq \;0$.

**Proposition** **3.** $\;d\omega_{t}/\;dt$ is the derivative of $\omega_{t}$ in the direction ω, with ω such that $\xi_{t}\left(\omega \right)=\chi_{t}\left(\rho_{1}\right)-\chi_{t}\left(\rho_{0}\right)$.

**Proof.**  From the definitions of $\chi_{\rho }$, $G_{\rho }$ and $A_{\sigma ,\tau },$ it follows

$$\begin{array}{ccc} \left(\pi \left(A\right)\Omega_{t},\chi_{t}\left(\omega_{\sigma }\right)\Omega_{t}\right) & = & \left(\pi \left(A\right)\Omega_{t},G_{t}^{-1}\pi \left(A_{\sigma_{t},\sigma }\right)\Omega_{t}\right)\\ & = & \int_{0}^{1}\;du\;\left(\pi \left(A\right)\Omega_{t},\pi \left(\rho_{t}^{u}A_{\sigma_{t},\sigma }\rho_{t}^{-u}\right)\Omega_{t}\right)\\ & = & \int_{0}^{1}\;du\;\left(\pi \left(\rho_{t}^{-u}A_{\sigma_{t},\sigma }\rho_{t}^{u}A\right)\Omega_{t},\Omega_{t}\right)\\ & = & D(\rho_{t}\left||\sigma \right)\rho_{t}\left(A\right)\\ & & +\int_{0}^{1}\;du\;\left(\pi \left(A\right)\Omega_{t},\pi \left(\rho_{t}^{u}\left(\log\sigma -\log\rho_{t}\right)\rho_{t}^{-u}\right)\Omega_{t}\right). \end{array}$$

Use this for $\sigma =\rho_{1}$ and for $\sigma =\rho_{0}$ and subtract. This gives

$$\begin{array}{ccc} \left(\pi \left(A\right)\Omega_{t},\left[\chi_{t}\left(\rho_{1}\right)-\chi_{t}\left(\rho_{0}\right)\right]\Omega_{t}\right) & = & \left[D(\rho_{t}\left|\right|\rho_{1})-D(\rho_{t}\left|\right|\rho_{0})\right]\rho_{t}\left(A\right)\\ & + & \int_{0}^{1}\;du\;\left(\pi \left(A\right)\Omega_{t},\pi \left(\rho_{t}^{u}\left(\log\rho_{1}-\log\rho_{0}\right)\rho_{t}^{-u}\right)\Omega_{t}\right)\\ & = & -\omega_{t}\left(H\right)\rho_{t}\left(A\right)\\ & + & \int_{0}^{1}\;du\;\left(\pi \left(A\right)\Omega_{t},\pi \left(\rho_{t}^{u}H\rho_{t}^{-u}\right)\Omega_{t}\right)\\ & = & -\omega_{t}\left(H\right)\rho_{t}\left(A\right)\\ & + & \int_{0}^{1}\;du\;\;\mathrm{Tr}\;\rho_{t}^{1-u}H\rho_{t}^{u}A\\ & = & \frac{\;d\;}{\;dt}\omega_{t}\left(A\right)\\ & = & f_{t,K}, \end{array}$$

with $K=\chi_{t}\left(\rho_{1}\right)-\chi_{t}\left(\rho_{0}\right)$. ☐

The following result shows that $\chi_{\rho }\left(\omega \right)$ is an affine coordinate in the case of the exponential connection.

**Proposition** **4.** *Let us be given a strictly positive density matrix ρ and a geodesic $t\mapsto \rho_{t}$ of the form (19). Then,*

(24) $$\begin{array}{ccc} \chi_{\rho }\left(\omega_{t}\right) & = & \left(1-t\right)\chi_{\rho }\left(\omega_{0}\right)+t\chi_{\rho }\left(\omega_{1}\right). \end{array}$$

**Proof.**  From (iii) of Theorem 5, it follows that for all A∈A

$$\begin{array}{ccc} \left(\pi \left(A\right)\Omega_{\rho },\chi_{\rho }\left(\omega_{t}\right)\Omega_{\rho }\right) & = & \frac{\;d\;}{\;ds}{|}_{s=0}\;\mathrm{Tr}\;\sigma_{s}A, \end{array}$$

with $\sigma =\rho_{t}$. The latter implies

$$\begin{array}{ccc} \sigma_{s} & = & \frac{1}{Z_{t}\left(s\right)}\exp\left(\log\rho +s(\log\rho_{t}-\log\rho )\right), \end{array}$$

where

$$\begin{array}{ccc} Z_{t}\left(s\right) & = & \;\mathrm{Tr}\;\exp\left(\log\rho +s(\log\rho_{t}-\log\rho )\right). \end{array}$$

Use Expression (19) to obtain

$$\begin{array}{ccc} \sigma_{s} & = & \frac{1}{Z_{t}\left(s\right)}\exp\left(\left(1-s\right)\log\rho +s(\left(1-t\right)\log\rho_{0}+t\log\rho_{1}-\zeta \left(t\right))\right). \end{array}$$

There follows

$$\begin{array}{ccc} \left(\pi \left(A\right)\Omega_{\rho },\chi_{\rho }\left(\omega_{t}\right)\Omega_{\rho }\right) & = & \int_{0}^{1}\;du\;\;\mathrm{Tr}\;\rho^{u}\left[-\log\rho +\left(1-t\right)\log\rho_{0}+t\log\rho_{1}-\zeta \left(t\right)\right]\rho^{1-u}A\\ & & -\frac{\;d\;}{\;ds}{|}_{s=0}Z_{t}\left(s\right)\omega_{\rho }\left(A\right)\\ & = & \left(1-t\right)\int_{0}^{1}\;du\;\;\mathrm{Tr}\;\rho^{u}\left[-\log\rho +\log\rho_{0}-\zeta \left(t\right)\right]\rho^{1-u}A\\ & & +t\int_{0}^{1}\;du\;\;\mathrm{Tr}\;\rho^{u}\left[-\log\rho +\log\rho_{1}-\zeta \left(t\right)\right]\rho^{1-u}A\\ & & -\frac{\;d\;}{\;ds}{|}_{s=0}Z_{t}\left(s\right)\omega_{\rho }\left(A\right)\\ & = & \left(1-t\right)\left(\pi \left(A\right)\Omega_{\rho },[\chi_{\rho }\left(\omega_{0}\right)\Omega_{\rho }\right)+t\left(\pi \left(A\right)\Omega_{\rho },\chi_{\rho }\left(\omega_{1}\right)]\Omega_{\rho }\right)\\ & & \frac{\;d\;}{\;ds}{|}_{s=0}Z_{0}\left(s\right)\omega_{\rho }\left(A\right)+\frac{\;d\;}{\;ds}{|}_{s=0}Z_{1}\left(s\right)\omega_{\rho }\left(A\right)\\ & & -\frac{\;d\;}{\;ds}{|}_{s=0}Z_{t}\left(s\right)\omega_{\rho }\left(A\right)\\ & = & \left(\pi \left(A\right)\Omega_{\rho },\left(1-t\right)\chi_{\rho }\left(\omega_{0}\right)+t\chi_{\rho }\left(\omega_{1}\right)\right). \end{array}$$

Because A∈A is arbitrary and $\Omega_{\rho }$ is cyclic and separating one concludes that Equation (24) holds. ☐

## 10. Modular Automorphisms

The quantum manifold M carries an additional structure, which is induced by the modular automorphism groups, one for each ρ∈M. In the commutative case, the automorphisms become trivial.

The Tomita–Takesaki theory [33] associates with each state $\omega_{\rho }$ in M a self-adjoint operator $\Delta_{\rho }$ on $H_{\pi }$, which is called the modular operator. The one-parameter group of unitary operators $\Delta_{\rho }^{it}$ defines a group of inner automorphisms of the algebra A. Indeed, for any *A* in A, the operator $\Delta_{\rho }^{it}\pi \left(A\right)\Delta_{\rho }^{-it}$ belongs again to π(A). In particular, it induces a group of transformations of the manifold M by mapping any state $\omega_{\sigma }$ onto the state $\omega_{\sigma ,t}$ defined by

$$\begin{array}{ccc} \omega_{\sigma ,t}\left(A\right) & = & \left(\Delta_{\rho }^{it}\pi \left(A\right)\Delta_{\rho }^{-it}\Omega_{\sigma },\Omega_{\sigma }\right). \end{array}$$

This group of transformations has $\omega_{\rho }$ as a fixpoint because $\Delta \Omega_{\rho }=\Omega_{\rho }$.

A useful property of the group of modular automorphims is the so-called *KMS condition*, named after Kubo, Martin and Schwinger. Given two elements *A* and *B* of A, the function F(t), defined by

$$\begin{array}{ccc} F(t) & = & \left(\pi \left(A\right)\Delta_{\rho }^{it}\pi \left(B\right)\Omega_{\rho },\Omega_{\rho }\right), \end{array}$$

has an analytic continuation in the complex plane such that

$$\begin{array}{ccc} F(t+i) & = & \left(\pi \left(B\right)\Delta_{\rho }^{it}\pi \left(A\right)\Omega_{\rho },\Omega_{\rho }\right). \end{array}$$

This property captures the essence of cyclic permutation under the trace and is helpful in the more general context when manipulating non-commuting pairs of operators.

## 11. Discussion

This paper reviews known and less known results of quantum information geometry. The Hilbert space is assumed to be finite-dimensional to avoid the technicalities coming with unbounded operators. They give rise to domain problems and require a specific choice of operator norm—see [23,28].

The present point of view differs from the usual one, which starts from the Hilbert space generated by the density matrices. Instead, the GNS-representation is used because it is more suited for later generalizations. The main goal of the present work is precisely to present those results for which one would like to find generalizations in the infinitely-dimensional case.

The manifold M of faithful quantum states can be parameterized in many ways. It is tradition to label each quantum state ω by a corresponding density matrix ρ. Here, the parameter-free approach of Pistone and coworkers [1,3,4,27] is followed. In particular, with each element $\omega_{\rho }$ of M is associated a chart centered at $\omega_{\rho }$. Two atlases are introduced. The atlas with the charts $\xi_{\rho }$, introduced in Section 4, is technically less complicated. It turns M into a Banach manifold. However, it is not linked in a straightforward manner with the Riemannian metric induced by Bogoliubov’s inner product. Therefore, another set of charts, denoted $\chi_{\rho }$, is introduced in Section 6. A link between the charts $\xi_{\rho }$ and $\chi_{\rho }$ is found in Proposition 3.

The dually affine connections are shortly mentioned in Section 8 and Section 9. In the case of the exponential connection, the charts $\chi_{\rho }$ provide affine coordinates.

## Appendix. The GNS-Representation of a Matrix Algebra

This appendix is added for convenience of the reader. Its content is well-known.

Choose an orthonormal basis of eigenvectors $\psi_{n}$, n=1,2,⋯,N, of the strictly positive density matrix ρ. It can be written as

$$\begin{array}{c} \rho =\sum_{n=1}^{N}p_{n}E_{n}\;\mathrm{where}\;E_{n}\;\mathrm{is}\mathrm{the}\mathrm{orthogonal}\mathrm{projection}\mathrm{onto}\;C\psi_{n}. \end{array}$$

Introduce now the vector $\Omega_{\rho }$ in H⊗H defined by

$$\begin{array}{c} \Omega_{\rho }=\sum_{n=1}^{N}\sqrt{p_{n}}\psi_{n}⊗\psi_{n}. \end{array}$$

It satisfies $\left|\right|\Omega_{\rho }{\left|\right|}^{2}=\sum_{n=1}^{N}p_{n}=1$. A short calculation shows that

$$\begin{array}{ccc} \;\mathrm{Tr}\;\rho A & = & (\left(A⊗I\right)\Omega_{\rho },\Omega_{\rho })\;\mathrm{for}\mathrm{all}\;A\in A. \end{array}$$

By assumption, all eigenvalues $p_{n}$ are strictly positive. Therefore, $||A\Omega_{\rho }\left|\right|=0$ implies A=0. This shows that $\Omega_{\rho }$ is separating. Let $E_{n,m}$ be the orthogonal matrix which maps $\psi_{m}$ onto $\psi_{n}$. It belongs to A and satisfies

$$\begin{array}{ccc} E_{n,m}\Omega_{\rho } & = & \sqrt{p_{m}}\psi_{n}⊗\psi_{m}. \end{array}$$

This shows that $A\Omega_{\rho }$ equals all of H⊗H. Hence, $\Omega_{\rho }$ is a cyclic vector for A⊗I. Because the GNS-representation is unique up to unitary equivalence, one concludes that A↦A⊗I, together with the Hilbert space H⊗H and the vector $\Omega_{\rho }$ is equivalent.

Note that the commutant of A⊗I equals I⊗A.

Introduce now an anti-linear operator *J* defined by

$$\begin{array}{c} J\left(A⊗I\right)\Omega_{\rho }=\left(I⊗A^{\prime }\right)\Omega_{\rho } \end{array}$$

with $A^{\prime }$ given by

$$\begin{array}{ccc} A^{\prime }\psi_{n} & = & \sum_{m}\left(A^{*}\psi_{m},\psi_{n}\right)\psi_{m}. \end{array}$$

From $\left|\right|A^{\prime }\psi_{n}\left|\right|=||A\psi_{n}\left|\right|$, it then follows that *J* is an isometry. A short calculation shows that for all A,B

$$\begin{array}{ccc} \left(J\left(A⊗I\right)\Omega_{\rho },J\left(B⊗I\right)\Omega_{\rho }\right) & = & (\left(B⊗I\right)\Omega_{\rho },\left(A⊗I\right)\Omega_{\rho }). \end{array}$$

Next, define an anti-linear operator *S* by $S\left(A⊗I\right)\Omega_{\rho }=\left(A^{*}⊗I\right)\Omega_{\rho }$. This is the modular conjugation operator. One verifies immediately that the conjugate *F* of *S* satisfies $F\left(I⊗A\right)\Omega_{\rho }=\left(I⊗A^{*}\right)\Omega_{\rho }$ for all *A*.

The modular operator Δ by definition equals $S^{*}S=FF^{*}$. Let us verify that $\Delta =\rho ⊗\rho^{-1}$. It suffices to show that $\rho^{-1/2}⊗\rho^{1/2}J=S$. One calculates

$$\begin{array}{ccc} \left(\rho^{-1/2}⊗\rho^{1/2}\right)J\left(A⊗I\right)\Omega_{\rho } & = & \left(\rho^{-1/2}⊗\rho^{1/2}\right)\left(I⊗A^{\prime }\right)\Omega_{\rho }\\ & = & \sum_{n}\sqrt{p_{n}}\left(\rho^{-1/2}⊗\rho^{1/2}\right)\psi_{n}⊗A^{\prime }\psi_{n}\\ & = & \sum_{n}\psi_{n}⊗\rho^{1/2}A^{\prime }\psi_{n}\\ & = & \sum_{m,n}\left(A^{*}\psi_{m},\psi_{n}\right)\psi_{n}⊗\rho^{1/2}\psi_{m}\\ & = & \sum_{m,n}\sqrt{p_{m}}\left(A^{*}\psi_{m},\psi_{n}\right)\psi_{n}⊗\psi_{m}\\ & = & \sum_{m}\sqrt{p_{m}}A^{*}\psi_{m}⊗\psi_{m}\\ & = & \left(A^{*}⊗I\right)\Omega_{\rho }\\ & = & S\left(A⊗I\right)\Omega_{\rho }. \end{array}$$

From ${\left(\rho A\right)}^{\prime }=\rho A^{\prime }$, it follows that J(ρ⊗I)=(I⊗ρ)J and hence

$$\begin{array}{c} S=\left(\rho^{-1/2}⊗\rho^{1/2}\right)J=J\left(\rho^{1/2}⊗\rho^{-1/2}\right). \end{array}$$

This implies that $\Delta =\rho ⊗\rho^{-1}$.
