# Correction: Naudts, J. Quantum Statistical Manifolds. *Entropy* 2018, *20*, 472

**DOI:** 10.3390/e20100796

**Published:** 2018-10-17

**Authors:** Jan Naudts

**Affiliations:** Departement Fysica, Universiteit Antwerpen, Universiteitsplein 1, 2610 Wilrijk Antwerpen, Belgium; jan.naudts@uantwerpen.be

## Abstract

Section 4 of “Naudts J. Quantum Statistical Manifolds. *Entropy* **2018**, *20,* 472” contains errors. They have limited consequences for the remainder of the paper. A new version of this Section is found here. Some smaller shortcomings of the paper are taken care of as well. In particular, the proof of Theorem 3 was not complete, and is therefore amended. Also, a few missing references are added.

**Theorem** **1.**

 

**Theorem** **2.**

 

## 1. Corrections in Section 3

The display on top of page 5 should read

$$\begin{array}{ccc} \left|\right|f_{\rho ,K}\left|\right| & = & \underset{A\in A}{\sup}\left\{f_{\rho ,K}\left(A\right):\;||A||\leq 1\right\}\\ & = & \underset{A\in A}{\sup}\left\{(\pi \left(A\right)K\Omega_{\rho },\Omega_{\rho }):\;||A||\leq 1\right\}\\ & = & {\left||\;|K\right|}^{1/2}\Omega_{\rho }{\left|\right|}^{2}\\ & \leq & {\left||\;|K\right|}^{1/2}{\left|\right|}^{2}=||K||. \end{array}$$

The operator *K* is replaced by |K| because *K* need not be positive.

The sentence ”This is a prerequisite for proving in the next Theorem that this map is the Fréchet derivative of the chart $\xi_{\rho }$.” should read ”This is a prerequisite for proving in the next Theorem that this map is the Fréchet derivative of the inverse of the chart $\xi_{\rho }$.”

The proof of the following Theorem is amended.

**Theorem** **3.** *The inverse of the map $\xi_{\rho }:\;M\mapsto B_{\rho }$, defined in Theorem 2, is Fréchet-differentiable at $\omega =\omega_{\rho }$. The Fréchet derivative is denoted $F_{\rho }$. It maps K to $f_{\rho ,K}$, where the latter is defined by (10).*

**Proof.**  Let $K=\xi_{\rho }\left(\omega_{\sigma }\right)$. One calculates

(11) $$\begin{array}{ccc} \left|\right|\omega_{\sigma }-\omega_{\rho }-F_{\rho }K\left|\right| & = & \underset{A\in A}{\sup}\left\{|\omega_{\sigma }\left(A\right)-\omega_{\rho }\left(A\right)-F_{\rho }K\left(A\right)|:\;||A||\leq 1\right\}\\ & = & \underset{A\in A}{\sup}\left\{|(\pi \left(A\right)\Omega_{\rho },\left[e^{K-\alpha (K)}-I-K\right]\Omega_{\rho })|:\;||A||\leq 1\right\}\\ & \leq & \left|\right|e^{K-\alpha (K)}-I-K\left|\right|\\ & \leq & |\alpha (K)|+o(||K-\alpha (K)||). \end{array}$$

Note that

$$\begin{array}{c} \left|\alpha \left(K\right)\right|\leq \log||e^{K}\left|\right|\leq \left||K|\right| \end{array}$$

and

$$\begin{array}{c} ||K-\alpha (K)||\leq 2||K||. \end{array}$$

In addition, if ||K||<1 then one has

$$\begin{array}{c} \alpha_{\rho }\left(K\right)\leq \log(1+||K\Omega_{\rho }{\left|\right|}^{2})\leq ||K\Omega_{\rho }{\left|\right|}^{2}. \end{array}$$

This holds because λ≤1 implies $\exp\left(\lambda \right)\leq 1+\lambda +\lambda^{2}$. One concludes that (11) converges to 0 faster than linearly as ||K|| tends to 0. This proves that $F_{\rho }K$ is the Fréchet derivative of $\xi_{\rho }\left(\omega_{\sigma }\right)\mapsto \omega_{\sigma }$ at σ=ρ. ☐

## 2. New Version of Section 4

Propositions 1 and 2 of [1] are not correct. This only has consequences for one sentence in the Introduction of [1] and for the results reported in Section 4 of [1]. The text in the Introduction “Next, an atlas is introduced which contains a multitude of charts, one for each element of the manifold. Theorem 4 proves that the manifold is a Banach manifold and that the cross-over maps are linear operators.” should be changed to “Next, an atlas is introduced which contains a multitude of charts, one for each element of the manifold. Theorem 4 proves that the manifold is a Banach manifold and that the cross-over maps are continuous.”

A new version of Section 4 follows below:

## 4. The Atlas

Following the approach of Pistone and collaborators [1,3,4,24], we build an atlas of charts $\xi_{\rho }$, one for each strictly positive density matrix ρ. The compatibility of the different charts requires the study of the cross-over map $\xi_{\rho_{1}}\left(\sigma \right)\mapsto \xi_{\rho_{2}}\left(\sigma \right)$, where $\rho_{1},\rho_{2},\sigma$ are arbitrary strictly positive density matrices.

Simplify notations by writing $\xi_{1}$ and $\xi_{2}$ instead of $\xi_{\rho_{1}}$, respectively $\xi_{\rho_{2}}$. Similarly, write $\Omega_{1}$ and $\Omega_{2}$ instead of $\Omega_{\rho_{1}}$, respectively $\Omega_{\rho_{2}}$, and $F_{1},F_{2}$ instead of $F_{\rho_{1}}$, respectively $F_{\rho_{2}}$.

**Proposition** **1.** *RETRACTED*

Continuity of the cross-over map follows from the continuity of the exponential and logarithmic functions and from the following result.

**Proposition** **2.** Fix strictly positive density matrices $\rho_{1}$ and $\rho_{2}$. There exists a linear operator Y such that for any strictly positive density matrix σ and corresponding positive operators $X_{1}$, $X_{2}$ in the commutant $A^{\prime }$ one has $X_{2}=YX_{1}Y^{*}$.

**Proof.**  Using the notations of the Appendix of [1], one has

$$\begin{array}{ccc} X_{i} & = & J_{i}\left(\rho_{i}^{-1/2}\sigma \rho_{i}^{-1/2}⊗I\right)J_{i}^{*},\;i=1,2. \end{array}$$

Note that the isometry *J* depends on the reference state with density matrix ρ. Therefore, it carries an index *i*. The above expression for $X_{i}$ implies that

$$\begin{array}{c} X_{2}=YX_{1}Y^{*}\;\mathrm{with}\;Y=J_{2}\left(\rho_{2}^{-1/2}\rho_{1}^{1/2}⊗I\right)J_{1}^{*}. \end{array}$$

 ☐

**Theorem** **4.** *The set M of faithful states on the algebra A of square matrices, together with the atlas of charts $\xi_{\rho }$, where $\xi_{\rho }$ is defined by Theorem 1, is a Banach manifold. For any pair of strictly positive density matrices $\rho_{1}$ and $\rho_{2}$, the cross-over map $\xi_{2}\circ \xi_{1}^{-1}$ is continuous.*

**Proof.**  The continuity of the map $X_{1}\mapsto X_{2}$ follows from the previous Proposition. The continuity of the maps $K_{1}\mapsto X_{1}$ and $X_{2}\mapsto K_{2}$ follows from the continuity of the exponential and logarithmic functions and the continuity of the function α. ☐

## 3. Corrections in Section 9

In the proof of Proposition 4, the symbol $\Omega_{\rho }$ is missing five times in obvious places. It has been added.

## 4. Added References

In the overview of papers devoted to the study of the quantum statistical manifold in the finite-dimensional case, the references [2,3] should be added. A quantum version of the work of Pistone and Sempi [4], alternative to [5], is found in [6]. Reference [7] to the work of Ciaglia et al. has been updated.
